# Problem Gambling Features and Gendered Gambling Domains Amongst Regular Gamblers in a Swedish Population-Based Study

**DOI:** 10.1007/s11199-014-0354-z

**Published:** 2014-02-12

**Authors:** Jessika Svensson, Ulla Romild

**Affiliations:** 1 Department of Health Science, Mid Sweden University, Östersund, Sweden; 2 Swedish National Institute of Public Health, Östersund, Sweden

**Keywords:** Gender, Regular gamblers, Problem gambling severity index (PGSI), Prevalence, Problem gambling

## Abstract

This study aimed to investigate, from a gender perspective, how different features of problem gambling present in men and women who gamble regularly in Sweden were distributed in four domains based on gambling type (chance or strategy) and setting (public or domestic). Problem gambling features were based on the nine items in the Problem Gambling Severity Index (PGSI). It was hypothesized that men and women gamble in different domains. Further, it was hypothesized that male gamblers overall experienced more problems with gambling than female gamblers, although in the same domains they would report the same level of problems. A further hypothesis predicted that regular female gamblers would experience more health and social problems and men would experience more financial difficulties. Interviews with a subsample of gamblers (*n* = 3191) from a Swedish nationally representative sample (*n* = 8179) was used to examine how features of problem gambling correspond with gender and the domains. Only the first hypothesis was fully supported. Men were more likely to participate in forms of gambling requiring strategy in a public setting, and women were more likely to participate in chance-based gambling in a domestic setting. Male and female gamblers had similar levels of problem gambling in the bi-variate analysis, but if controlling for age and gambling in multiple domains, women were more at risk than men. Additionally, men and women presented similar health and economic situations. The differences between male and female gamblers in Sweden have implications for research and prevention.

## Introduction

Gambling is generally permitted and a popular leisure activity in Sweden (Blomqvist 2009; Statens folkhälsoinstitut 2010) as well as in other European countries including United Kingdom (Wardle et al. 2011), Finland (Jaakola 2009), the Netherlands (Goudriaan et al. 2009) and Spain (Becona 2009). In Sweden like most parts of Europe, the accessibility of gambling opportunities has increased due to introduction of new media (Meyer et al. 2009). Findings from population studies in Canada, United States and United Kingdom, (Welte et al. 2006; el-Guebaly et al. 2006; Wardle et al. 2011) have led researchers to suggest that, similar to drug or alcohol use, participation in gambling ranges along a continuum from non-consumption to controlled consumption to uncontrollable consumption, which may lead to severe social, health and economic problems (Castellani 2000; Shaffer and Kidman 2004).

Several gambling researchers suggest that genders should be analyzed separately to avoid the risk of omitting gender effects (Afifi et al. 2010; Blanco et al. 2006). Research exploring diverse female gambling patterns and motives is scarce (Cousins and Witcher 2004; Holdsworth et al. 2012; Trevorrow and Moore 1999) and most existing research has been through clinical studies of problem gamblers, not general regular gamblers (Shaffer et al. 2004). Sex role socialization contributes to opportunities, motives, and the development of skills, all of which may influence both interest and participation in gambling activities (Holdsworth et al. 2012; Stevens and Young 2010). Therefore, gambling is an arena for expression and validation of masculinities and femininities (Svensson et al. 2011). The implications of gender on gambling behavior and motives need to be understood to prevent problem gambling and for breaking masculine norms in gambling, thereby changing the unequal gender order.

### Gender Equality in Sweden

A Swedish study has shown that men in Sweden display a higher level of social dominance orientation (SDO) than women, which favors group dominance and opposes equality among groups in society (Zackrisson 2008). The only exception is found in situations and organizations that are defined by their democratic and equality features, where women dominate. Zackrisson (2008) questions the levels of SDO in women in male dominated associations. It is possible that women in male dominated, masculine domains conform to SDO. However, they may also be more opposed to SDO. When this is transferred to the context of gender and gambling, it may be relevant in understanding why gender stereotypes appear to remain in gambling and how women who gamble in masculine domains view the gendered aspect of gambling. Even if there are no legal obstacles for women to gamble, economic and social factors may act as constraints for women to enter specific domains of gambling.

According to the Global Gender Gap Report 2012, Sweden is one of the world leaders in gender equality regarding economics, politics, education and health (Hausmann et al. 2012). Sweden is known as a welfare state, which tries to overcome gender-based inequality through implementation of policies which target gender socialization and traditional gender roles (Bambra et al. 2009). However, even with these policies the gender gap in income in Sweden has not decreased in 30 years. The most important reason for this lack of decrease in income gap is women’s responsibilities for childcare and domestic tasks (Angelov et al. 2013). Regardless of class or positions in the workplace, women in Sweden, as in most countries, are generally still primarily responsible for the private sphere of caring for the family and the home (Statistic Sweden 2011). This is relevant to gambling when understanding gambling behavior of women and how women and men experience features of problem gambling differently, such as feelings of guilt and criticism.

In 2012, compared to other European countries Sweden had higher rate of female employment and was, together with Finland, Denmark and Belgium, one of the four countries in the European Union which had a balanced representation with at least 40 % of each gender (European Commission 2013). Studies on gender role attitudes have shown that Swedish respondents generally disapprove of work specialization according to gender. Female employment is widely accepted, men are increasingly expected to contribute to household and family work, and fewer tasks are regarded as either “male” or “female” (European Parliament 2013). However, women in gainful employment and who generally strive to live gender-equal lives tend to be subject to greater stress than women with more traditional gender roles, which may cause the former health problems (Nordenmark 2008). Life time prevalence rate for violence against women aged 15 and above in Sweden was almost 50 % in the latest prevalence study on violence against women in Sweden (Lundgren et al. 2001). One-third of women in Sweden report that they refrain from going out due to fear of assault, compared to less than 9 % of men (Swedish National Institute of Public Health 2013). This may impact women’s preferences of gambling venues and settings.

### Gambling Domains

Gambling is historically a male-dominated activity, and not all types of gambling are equally accessible or culturally acceptable for women (Casey 2006; Hing and Breen 2001). Gavriel-Fried et al. (2010) showed that Israeli female pathological gamblers with children attempted to juggle between caring gender roles and gambling in order to minimize social costs. Research on female gamblers in the United States showed that women had less free time than men and that their free time was only available in smaller time periods (Winn and Heeter 2009). This finding is consistent with Swedish surveys showing that women spend more time on housework than men and report less leisure time than men (Statistic Sweden 2012). This may affect women’s access to gambling.

In Australia, UK and in the US, men appear to be more engaged in Internet gambling, sports betting and horse racing while women prefer lotteries, raffles, slot/gambling machines and bingo (Department of Justice Victoria 2011; Ipsos-Reid and Gemini Research 2008; Wardle et al. 2011). In Sweden, buying lottery tickets is a mainstream gambling activity for both genders. Over half of all Swedish men and women aged 16–84 bought lottery tickets during the last year. However, there have been, and still are, significant gender differences in gambling behavior. Men gamble more frequently than women (Abbott et al. 2004; Svensson et al. 2011) and also bet more money than women (Statens folkhälsoinstitut 2010). In 2008/2009, 28 % of the male population surveyed reported that they had gambled during the last week. The corresponding figure for women was 19 %. Men gambled more than women in all forms of gambling except for lotteries and bingo. The largest differences between men and women in the gambling prevalence rate are for casino games (men 13 %; women 3 %) and poker (men 17 %; women 4 %) (Statens folkhälsoinstitut 2010).

#### Games of Chance and Strategy

In the present paper, we refer to studies that avoid treating gambling as a homogeneous entity but try to deconstruct and analyze gambling forms according to their relation to characteristics such as “chance” and “skill” (strategy) (Bjerg 2010). Here, a gender aspect exists. Amongst other studies, as the Swedish population study (Statens folkhälsoinstitut 2010), two studies from United States has shown that men appear to be attracted to gambling that involves competing with and against others, whereas women generally prefer monotonous forms of gambling that entail playing against a house instead of other players (Burger et al. 2006; Nower and Blaszczynski 2006).

Stevens and Young (2010) found empirical support for the relevance of skill/strategy and chance as a conceptual framework by conducting a factor analysis based on the activities of previous-year gamblers in the Northern Territory of Australia. Svensson et al. (2011) extended the research of Stevens and Young (2010) by using their approach in examining gambling in Sweden, based on the idea that forms of gambling contain features of strategy and chance.

#### Domestic and Public Domains of Gambling

Svensson et al. (2011) supplemented the approach of strategy and chance with Casey’s (2006) concept of domestic and public gambling. According to this concept, women and femininity are associated with domestic gambling (i.e. gambling at home or in a home environment), through games of chance that are easy to learn and incorporate into everyday life. Although the gender role theory has been criticized as being outdated by some gambling researchers like Delfabbro (2009) when he discusses the Australian context, men are still associated with, and directed to, the public sphere and women are still associated with, and directed to, the private sphere and excluded from the public sphere in Sweden (Gíslason and Eydal 2011). Therefore, the dichotomy of public and private spheres is still relevant and was used in this study to examine gendered leisure constraints in gambling.

### Gambling-Related Problems

The structural composition of different forms of gambling can significantly affect the ways that gambling problems develop and present (Bjerg 2010). The increased risk of problem gambling for men is not surprising as men, in general, gamble more than women and are more likely to use forms of gambling significantly associated with a high risk of problem gambling (Holdsworth et al. 2012). A review of over 200 populations studies on problem gambling showed that gambling forms including gambling machines, poker, bingo, casino games and Internet gambling are in general more associated with the risk of problem gambling than others (Williams et al. 2012). The effect of adding gambling behavior to regression models that investigate problem gambling is noteworthy. In some studies, such as in the first wave of the Swedish longitudinal study and the South African prevalence study, being male was no longer a significant predictor for problem gambling after controlling for gambling behavior (Ross et al. 2010; Statens folkhälsoinstitut 2010). Notably, some places, such as Manitoba, Canada, report no gender differences; both men and women are equally likely to gamble and to be problem gamblers (Lemaire et al. 2008). This and the increase in women seeking help in Australia has been partly explained by the normalization, wide acceptance and availability of gambling machines (Delfabbro 2009; Holdsworth et al. 2012; Moore et al. 2011).

The proportion of problem gamblers in Sweden appears to be consistent at just over 2 % of the total of the population between 16–84 years, with 3.2 % among men and .5 % among women (Svensson et al. 2011). The highest proportion of problem gambling is found among men aged 18–24 where 1 in 10 has a gambling problem. In the oldest age group (65–84 years) the proportion of problem gambling is higher among women than men (Statens folkhälsoinstitut 2010). Therefore, gambling participation and age may act as important variables when examining problem gambling among male and female Swedish regular gamblers.

The Swedish help line for problem gamblers is predominantly used by men. Over 80 % of the 500–1100 annual calls over the last 4 years have been made by men (Statens folkhälsoinstitut 2011). These data are similar to the gender division of those seeking gambling treatments in Sweden: 83.5 % men (Carlbring et al. 2010). In 2004–2005, the proportion of men seeking Internet-based treatment was 94 % (Carlbring et al. 2012).

Gambling-related problems range from economic hardship to health problems. Wenzel and Dahl (2009) conducted a critical review of the literature which included 28 papers concerning the clinical characteristics of pathological gamblers. Problem gamblers of both genders demonstrated financial problems and legal problems, but men reported more criminal histories (Wenzel and Dahl 2009). They also concluded that there was strong evidence for a higher comorbidity of anxiety and mood disorders in female problem gamblers and for a higher comorbidity of alcohol abuse in male problem gamblers.

This study aimed to investigate how different features of problem gambling in men and women who gamble regularly are distributed and presented in different domains from a gender perspective. Based on the knowledge presented in the sections above, gambling was divided into four domains according to their characteristics in terms of features (strategy or chance) and setting (domestic or public).

### Hypotheses

As mentioned, gender differences exist in gambling as well as problem gambling. Women appear to gamble on forms of gambling that are characterized by chance, while men seem to be more involved in games of strategy. Extensive evidence also states that public spheres are viewed as masculine and the domestic environments as feminine. We hypothesized that men and women who gamble regularly are involved in different domains, with women more involved in the chance-domestic domain and men dominate the other domains (chance-public, strategy-domestic and strategy-public) (Hypothesis 1). In addition, we predicted that the chance-domestic domain would be significantly less associated with problem gambling (Hypothesis 2).

Evidence suggests that more men than women are problem gamblers; that men and women who gamble in similar ways experience the same levels of problem gambling and that men and women who are defined as problem gamblers experience different types of gambling-related problems. Therefore, we hypothesized that male gamblers overall experience more problems with gambling than female gamblers (Hypothesis 3a). However, we added the prediction that women and men within the same gambling domains report the same level of problems with gambling (Hypothesis 3b). Further, we predicted that women in all domains experience more gambling-related health problems (Problem Severity Gambling Index, PGSI, 6), experience more criticism for their gambling (PGSI 7) if they gamble in a public environment, and feel more guilt (PGSI 9) than men who gamble in the same domains, except for the chance-domestic domain (Hypothesis 4a). In addition, we predicted that men in all domains, except from the domain of chance-domestic would be more likely to answer affirmative for all items regarding financial problems (PGSI 1, 2, 3, 4, and 8) (Hypothesis 4b).

## Method

### Sample and Data Collection

The sample of regular gamblers in the current study was a subsample from the first wave of the Swedish longitudinal gambling study (Swelogs), a longitudinal population study on gambling and health. The primary method of data collection involved computer-supported telephone interviews conducted by Statistics Sweden from November 2008 to April 2009. To reduce attrition, questionnaires were sent with two reminders to non-respondents.

The sample frame population consisted of persons aged between 16 and 84 years from the total population register. Sweden has unique register databases that contain information regarding the population. For all inhabitants, most vital life events (such as births, marriages, children, relocation, migration, salaries, unemployment allowances, educational levels and grades) are registered in different population registers. All of these registers are available to researchers. The data from the interviews were supplemented by register data, such as the Longitudinal Integration Database for Health Insurance and Labour Market Studies (LISA, formerly LOUISE).

A stratified simple random sample of 15,000 was drawn from the frame population. Gender, age group, and estimated risk of problem gambling were used for stratification. The estimated risk was calculated from register variables according to results of a pilot study conducted in the spring of 2008. Therefore the sample had an overrepresentation of youth, people born outside of Sweden and recipients of social welfare or family members of social welfare recipients. Only unweighted data were used. The unweighted response frequency was 57.3 %, *n* = 8,179 (Table 1).

**Table 1:** Distribution of socio-demographic variables within the total sample and in regular gamblers according to gender

		Men (total sample *n* = 4091)	Women (total sample *n* = 4076)	Men (regular gamblers) *n* = 2048	Women (regular gamblers) *n* = 1143
		n of population (%)	n of population (%)	n of population (%)	n of population (%)
Age (years)		***χ*** ^**2**^_**(2)**_ **= 71.1,** ***p*** **< .001**		***χ*** ^**2**^_**(2)**_ **= 23.7,** ***p*** **< 001**	
	16–24	1677 (44.0)	1677 (43.0)	774 (37.8)	370 (32.4)
	25–44	1162 (27.2)	1351 (34.7)	567 (27.7)	410 (35.9)
	45−	1230 (28.8)	868 (22.3)	707 (34.5)	363 (31.8)
Level of education		*χ* ^2^_(2)_ = .91, *p* = .634		*χ* ^2^_(2)_ = 1.32, *p* = .581	
	Low	1750 (45.2)	1618 (45.0)	746 (39.3)	401 (37.2)
	Intermediate	1355 (35.0)	1235 (34.4)	790 (41.6)	466 (43.2)
	High	765 (19.8)	741 (22.3)	363 (19.1)	212 (19.6)
Family situation		***χ*** ^**2**^_**(3)**_ **= 359.9,** ***p*** **< .001**		***χ*** ^**2**^_**(3)**_ **= 109.5,** ***p*** **< .001**	
	Single without children	2256 (53.3)	2119 (54.8)	928 (45.7)	483 (42.7)
	Single with children	61 (1.4)	411 (10.6)	25 (1.2)	97 (8.6)
	Married without children	1247 (29.4)	801 (20.7)	690 (34.0)	325 (28.7)
	Married with children	671 (15.8)	537 (13.9)	389 (19.1)	226 (20.0)
Living in a larger city		*χ* ^2^_(1)_ = 34.9, *p* < .001		*χ* ^2^_(1)_ = 1.71, *p* = .191	
	Yes	707 (16.6)	845 (21.7)	284 (13.9)	178 (15.6)
	No	3561 (83.4)	3049 (78.3)	1763 (86.1)	965 (84.4)
Country of birth		***χ*** ^**2**^_**(1)**_ **= 408.7,** ***p*** **< .001**		***χ*** ^**2**^_**(1)**_ **= 89.1,** ***p*** **< .001**	
	Sweden	3483 (81.6)	2395 (61.5)	1753 (85.6)	821 (80.7)
	Outside Sweden	786 (18.4)	1501 (38.5)	295 (14.4)	322 (28.2)
Living on social welfare		***χ*** ^**2**^_**(1)**_ **= 908.7,** ***p*** **< .001**		***χ*** ^**2**^_**(1)**_ **= 204.0,** ***p*** **< .001**	
	Yes	373 (11.4)	1348 (45.7)	163 (9.4)	304 (31.0)
	No	2887 (88.6)	1599 (54.3)	1568 (90.6)	678 (69.0)

Differences between men and women, in the total sample and regular gambler respectively were tested by Pearson’s chi square test of independence

Pearson’s chi-square tests of independence with degrees of freedom within parenthesis. Bold figures indicate statistical significance

Every participant who gambled at least 6 times a year was included in the final sample for this study. There was a total of 3,191 persons, 64.2 % men (*n* = 2,048) and 35.8 % women (*n* = 1143). The mean age of the participants was 38.47 years for men (SD 20.66) and 38.17 for women (SD: 19.00). To be a regular gambler, the respondent had to have gambled at least twice a month in any gambling form. Overall, regular gamblers were more often men, Swedish born, married with or without children, and aged 45 or older, compared with the total population.

The bi-variate analyses found significant differences between men and women in the sample of regular gamblers. Women were more likely than men to be aged 25–44 while men were more likely than women to be younger than 25 or older than 45 (*p* < .001). In the total sample, women were more likely to live in a larger city than men (*p* < .001), but no such differences were found in regular gamblers (*p* = .191). Women were much more likely to receive social welfare or to have a family member receiving social welfare than men, in the total sample and in the sample of regular gamblers (*p* < .001 for all analyzes). The proportion of women born outside Sweden who were regular gamblers was larger than that of men born outside Sweden who were regular gamblers. With regard to education, no significant differences between the genders were observed in neither the total population (*p* = .634) nor the sample of regular gamblers (*p* = .581).

### The Four Gambling Domains and Gender Differences Regarding Social-Demography in Them

Four gambling domains were created based on the characteristics of each individual gambling form. Initially gambling forms were divided into two categories based on their basic orientation as either strategy (skill and competition) or chance (luck). Strategy and chance gambling forms were further divided based on their setting into either a *domestic* or *public* place. Appendix 2. Therefore, a person who gambled at least 6 times a year in more than one gambling domain was counted more than once in the analysis. All gambling forms were not easy to categorize. An exploratory factor analysis was undertaken and used as support when categorization by theory was difficult. For example, lottery tickets bought at an agent’s store could be played in the store, at home, or any other location. In this study, lotteries (besides tombolas) were included in the domain chance-domestic.

In all domains the proportions of single parents, recipients of social welfare, and people born outside Sweden were larger among female regular gamblers among male regular gamblers. There were no gender differences regarding the level of education or whether the participants lived in larger cities in any domain. There were no age differences between men and women in the public domains. In the domain of strategy-domestic men were younger and women were generally older (Table 2).

**Table 2:** Distribution of socio-demographic variables in the four gambling domains for men and women

		Chance-public (men *n* = 692)	Chance-public (women *n* = 280)	Strategy-domestic (men *n* = 715)	Strategy-domestic (women *n* = 90)	Chance-domestic (men *n* = 1,493)	Chance-domestic (women *n* = 1,018)	Strategy-public (men *n* = 845)	Strategy-public (women *n* = 195)
Age (years)		*χ* ^2^_(2)_ = 2.54, *p* = .281		***χ*** ^**2**^_**(2)**_ **= 7.54,** ***p*** **= .023**		***χ*** ^**2**^_**(2)**_ **= 21.0,** ***p*** **< .000**		*χ* ^2^_(2)_ = 1.18, *p* = .553	
	Mean age (SD)	39.0 (20.0)	40.0 (19.6)	**33.9 (17.9)**	**38.4 (18.5)**	**41.6 (20.7)**	**38.9 (18.9)**	34.5 (19.3)	35.6 (19.8)
	16–24	240 (34.7)	83 (29.6)	315 (44.1)	26 (28.9)	445 (29.8)	306 (30.1)	396 (46.9)	83 (42.6)
	25–44	206 (29.8)	94 (33.6)	229 (32.0)	37 (41.1)	443 (29.7)	380 (37.3)	211 (25.0)	53 (27.2)
	45−	246 (35.5)	103 (36.8)	171 (23.9)	27 (30.0)	605 (40.5)	332 (32.6)	238 (28.3)	59 (30.3)
Level of education		(*χ* ^2^_(2)_ = .65, *p* = .724)		(*χ* ^2^_(2)_ = 1.96, *p* = .375)		(*χ* ^2^_(2)_ = 1.56, *p* = .458)		(*χ* ^2^_(2)_ = .07, *p* = .967)	
	Low	247 (38.2)	94 (36.3)	249 (36.9)	26 (30.6)	453 (33.0)	341 (35.5)	390 (48.1)	88 (47.1)
	Middle	310 (47.9)	124 (47.9)	304 (45.0)	45 (52.9)	630 (45.9)	425 (44.2)	305 (37.6)	72 (38.5)
	High	90 (13.9)	41 (15.8)	122 (18.1)	14 (16.5)	290 (21.1)	195 (20.3)	116 (14.3)	27 (14.4)
Family situation		***χ*** ^**2**^_**(3)**_ **= 20.9,** ***p*** **< .001**		***χ*** ^**2**^_**(3)**_ **= 50.2,** ***p*** **= .001**		***χ*** ^**2**^_**(3)**_ **= 107.0,** ***p*** **= .001**		***χ*** ^**2**^_**(3)**_ **= 35.9,** ***p*** **= .001**	
	Single without children	310 (45.1)	121 (43.8)	345 (48.7)	39 (43.8)	567 (38.3)	412 (40.8)	429 (51.1)	97 (50.8)
	Single with children	8 (1.2)	17 (6.2)	8 (1.1)	12 (13.5)	16 (1.1)	93 (9.2)	9 (1.1)	16 (8.4)
	Married or coupled without children	241 (35.0)	82 (29.7)	205 (28.9)	19 (21.3)	573 (38.7)	297 (29.4)	255 (30.4)	50 (26.2)
	Married or coupled with children	129 (18.8)	56 (20.3)	151 (21.3)	19 (21.3)	324 (21.9)	207 (20.5)	147 (17.5)	28 (14.7)
Living in a larger city		*χ* ^2^_(1)_ = .75, *p* = .387		*χ* ^2^_(1)_ = 3.51, *p* = .061		*χ* ^2^_(1)_ = 3.51, *p* = .61		*χ* ^2^_(1)_ = .03, *p* = .871	
	Yes	605 (87.4)	239 (85.4)	597 (83.5)	82 (91.1)	1293 (86.7)	855 (84.0)	736 (87.1)	169 (86.7)
	No	87 (12.6)	41 (14.6)	118 (16.5)	8 (8.9)	199 (13.3)	163 (16.0)	109 (12.9)	26 (13.3)
Country of birth		***χ*** ^**2**^_**(1)**_ **= 20.9,** ***p*** **= .001**		***χ*** ^**2**^_**(1)**_ **= 15.4,** ***p*** **= .001**		***χ*** ^**2**^_**(1)**_ **= 111.1,** ***p*** **= .001**		***χ*** ^**2**^_**(1)**_ **= 6.7,** ***p*** **= .010**	
	Sweden	604 (8.3)	211 (75.4)	592 (82.8)	59 (65.6)	1307 (87.5)	719 (70.6)	733 (86.7)	155 (79.5)
	Outside Sweden	88 (12.7)	69 (24.6)	123 (17.2)	31 (34.4)	186 (12.5)	299 (29.4)	112 (13.3)	40 (20.5)
Social welfare		***χ*** ^**2**^_**(1)**_ **= 48.4,** ***p*** **= .001**		***χ*** ^**2**^_**(1)**_ **= 43.2,** ***p*** **= .001**		***χ*** ^**2**^_**(1)**_ **= 187.9,** ***p*** **= .001**		***χ*** ^**2**^_**(1)**_ **= 36.7,** ***p*** **= .001**	
	No	559 (87.)	162 (67.8)	540 (87.9)	50 (60.2)	1222 (91.7)	622 (69.3)	596 (90.7)	104 (72.2)
	Yes	77 (12.1)	77 (32.2)	74 (12.1)	74 (39.8)	111 (8.3)	276 (30.7)	61 (9.3)	40 (27.8)

Differences between men and women within same domains were tested by Pearson’s chi square test

Pearson’s chi-square tests of independence with degrees of freedom within parenthesis. Bold figures indicate statistical significance

### Measures

Education was separated into three categories based on the information from the register: low, intermediate and high. A low level of education was synonymous with completion of elementary school, an intermediate level of education with completion of upper secondary school and a high level of education with a degree from a university or tertiary institution. Ethnicity was analyzed using the register variable of “born in Sweden” or “born outside Sweden”. Family situation was divided into the categories single without children, single with children, married or coupled without children and married or coupled with children. All respondents were also categorized if they lived in one of Sweden’s three largest cities (Stockholm, Gothenburg and Malmö).

### Problem Gambling

PGSI is one of several instruments used to measure problem gambling in population surveys and was developed in response to criticism of other, more diagnostic instruments (Wynne and Ferris 2001). PGSI has become the gold standard in population-based research in many countries (Currie et al. 2010; Jackson et al. 2009) such as Canada (Wiebe et al. 2006; Volberg et al. 2006), Australia (Department of Justice Victoria 2011), Iceland (Olason and Gretarsson 2009), and United Kingdom (Wardle et al. 2010). Swelogs used PGSI to adopt a public health perspective and in order to be able to compare the results with international longitudinal studies, including the Victorian Gambling Study in Australia and the Leisure, Lifestyle and Lifecycle Project in Canada. PGSI consists of nine questions:

- PGSI 1. Have you bet more than you could afford to lose?
- PGSI 2. Have you needed to gamble with larger amounts of money to get the same feeling of excitement?
- PGSI 3. When you gambled, did you come back another day to get the money back?
- PGSI 4. Have you borrowed or sold anything to get money to gamble?
- PGSI 5. Have you felt that you might have a problem with gambling?
- PGSI 6. Has gambling caused you any health problems, including stress or anxiety?
- PGSI 7. Regardless of whether you think it was true, have people criticized your betting or told you that you have a gambling problem?
- PGSI 8. Has your gambling caused any financial problems for you or your household?
- PGSI 9. Have you felt guilty about the way you gamble or what happens when you gamble?

PGSI is an ordinal scale with four steps where the participant scores the following for each response: never = 0, sometimes = 1, most of the time = 2, almost always = 3. The maximum possible score is 27. The PGSI-population is usually divided into four groups: no problems (0), low risk (1–2), moderate risk (3–7) and problem gambling (8+). In some analyses in this study moderate risk and problem gambling were merged (PGSI 3+) as the statistical power would have otherwise been too weak. (Appendix 1).

Criticism of PGSI includes that it is too similar to diagnostic instruments and may not be applicable for a diverse population, or for use in gender analyses (Svetieva and Walker 2008). Stevens and Young (2010) argue that a gender-specific instrument is required. Maitland and Adams (2007) examined the factor structure of PGSI based on three independent datasets. They concluded that eventual gender differences based on PGSI are inappropriate because they may be the result of differing models between men and women (Maitland and Adams 2007). However, thresholds between response categories for each item within PGSI were the same for both genders, indicating that PGSI items perform similarly for men and women (Boldero and Bell 2011; Currie et al. 2010). Orford et al. (2010) found that female problem gambling was relatively under-estimated in the British prevalence survey when they compared the Diagnostic and Statistical Manual of Mental Disorders, fourth edition (DSM-IV) with PGSI. The prevalence of problem gambling for women was estimated as .2 % according to the DSM-IV scale, but only .1 % according to PGSI. In Swelogs, South Oakes Gambling Screen Revised (SOGS-R) and PGSI were used and in accordance with the findings of Orford et al. (2010), PGSI tended to relatively under-estimate female problem gambling compared to SOGS-R. This is further discussed in the limitations section below.

In our sample, Cronbach’s alpha was .82, with a mean value of .62 (SD 1.74) for men and women. For men, Cronbach’s alpha was .83 (SD 1.75) and for women, it was .82 (1.73).

Ethical approval was given by the Examination Board for Ethical Research at the Umeå Regional Ethical Review Board in Sweden.

## Results

### Participation in the Four Gambling Domains (Hypothesis 1)

Our first hypothesis predicted that women who gambled regularly are more involved in the domain chance-domestic and men dominate the other domains. We examined this hypothesis by four bi-variate analyses of participation in the different domains for women and men presented as proportions (e.g. percentages with Pearson’s chi-square tests of independence to test for significance). Our findings supported the hypothesis. Although participation in the chance-domestic domain was the most common among both genders, this was the only domain in which the proportion of participants among those women who were regular gamblers was higher than the proportion of participants among those men who were regular gamblers: with 89 % of the women and 73 % of the men (*p* < .001). A total of 71 % of men and 29 % of women gambled in the chance-public domain, including gambling machines in pubs and casinos as well as bingo at bingo halls (*p* < .001). Both gambling domains featuring strategy were heavily dominated by men; less than 1 in every 5 gambler was a woman in the strategy domains (*p* < .001). Notably, men who gambled regularly gambled in multiple domains more often than women who gambled regularly; with an average of two domains compared with 1.4 for women (*p* < .001) (Table 3).

**Table 3:** Number and proportion of participation for men and women within the four gambling domains

	Men (*n* = 2048) who had gambled in the domain, n (%)	Women (*n* = 1143) who had gambled in the domain, n (%)
Chance-public	692 (33.8)	280 (24.5)
Strategy-domestic	715 (34.9)	90 (7.9)
Chance-domestic	1493 (72.9)	1018 (89.1)
Strategy-public	845 (41.3)	195 (17.1)

### Problem Gambling in the Four Domains (Hypotheses 2, 3a, 3b)

Hypothesis 2 predicted that the domain of chance-domestic was significantly less associated with problem gambling among both men and women and, further, that male regular gamblers in total, were more likely to be problem gamblers than female regular gamblers (Hypothesis 3a). To test this hypothesis and the hypotheses 3a–b, one logistic regression analysis was performed in each domain to examine how gambling in the domain affected the probability of being a problem gambler according to PGSI for men and women separately (reported as odds ratios, ORs). 95 % confidence intervals (CIs) were computed for the ORs. Logistics models were tested by the Hosmer Lemeshow goodness-of-fit testing model. Further, two additional logistic regression analyses in each domain controlled for age and age and gambling in more than one domain, respectively. Hypothesis 3a was not supported by our results. No significant gender difference was found between the overall proportion of problem gambling among men and women who gambled regularly. A total of 7.9 % of the women scored as problem gamblers according to the PGSI (*n* = 90) compared with 7.5 % of the men (*n* = 154) (OR 1.1, CI .8–1.4). However, after controlling for age and gambling in other domains, women were significantly more likely to be problem gamblers (OR 1.4, CI 1.0–1.8).

Table 4 shows the results from testing hypothesis 3b. Of particular note is the high odds ratio in the chance-public domain which indicates an association between regular gambling and an increased risk of problem gambling for women. This domain was also associated with problem gambling for regular male gamblers, but the relationship disappeared after controlling for age and gambling in other domains. The confidential intervals show that there were no differences between genders regarding the proportions and odds ratios for problem gambling within the domains. Hypothesis 3b was supported.

**Table 4:** Number of male and female problem gamblers within the four gambling domains and multivariate models for odds ratios (OR) regarding problem gambling for men and women within the four domains^a^

Domain	Gender (n)	Problem gambling n (%)	OR crude (CI 95 %)	OR control for age (CI 95 %)	OR control for both age and gambling in other domains (CI 95 %)
Chance-public	Men (692)	85 (12.3)	**2.6 (1.9–3.6)**	**2.9 (2.1–4.1)**	1.5 (.9–2.4)
	Women (280)	45 (16.1)	**3.5 (2.2–5.4)**	**3.8 (2.4–5.9)**	**3.1 (1.6–5.7)**
Strategy-domestic	Men (715)	94 (13.1)	**3.2 (2.3–4.5)**	**2.8 (2.0–3.9)**	**2.0 (1.4–2.8)**
	Women (90)	13 (14.4)	**2.1 (1.1–4.0)**	**2.2 (1.2–4.2)**	1.2 (.6–2.5)
Chance-domestic	Men (1493)	103 (6.9)	**.5 (.3–.7)**	.7 (.5–1.2)	**.4 (.1–.7)**
	Women (1018)	72 (7.1)	.7 (.5–1.3)	.7 (.3–1.4)	.5 (.3–1.1)
Strategy-public	Men (845)	90 (10.7)	**2.3 (1.9–2.8)**	**2.0 (1.3–3.4)**	1.4 (.9–1.8)
	Women (195)	27 (13.8)	1.7 (.8–2.8)	1.8 (.9–3.6)	1.0 (.7–2.3)

Bold figures indicate statistical significance

^a^Compared to men

The strategy domains were associated with problem gambling for regular male gamblers. However, the association became weaker in when the variables for gambling in other domains and age were added. In the strategy-domestic domain the relation became weaker when controlling for age and it disappeared when controlling for gambling in other domains and age.

For men, the chance-domestic domain differed from the other domains by being associated with less probability for problem gambling in the bi-variate analysis. This relationship ceased to exist when controlling for age, but was present when controlled for gambling in other domains. However, for women, this domain was not associated with less risk for problem gambling, which suggests that hypotheses 2; that the domain of chance-domestic is significantly less associated with problem gambling among both men and women, was only partially supported.

### PGSI-Items and the Four Domains (Hypothesis 4a, 4b)

The last two hypotheses regarded the distribution of affirmative answers to the different PGSI-items among men and women in different domains. Hypotheses 4a predicted that women in all domains experience more health related problems (PGSI 6), experience more criticism for their gambling (PGSI 7) if they gamble in a public environment and feel more guilt (PGSI 9) than men in the same domains, except for the chance-domestic domain. In addition, hypotheses 4b predicted that men in all domains except for the chance-domestic are more likely than women to answer affirmative on all items regarding financial problems (PGSI 1, 2, 3, 4 and 8). We used the ordinal scale for PGSI instead of the merged dichotomy variable for each item and we used the Mann–Whitney test, which is a non-parametric test, for independent samples to test the significance. Our findings paralleled the findings of the overall PGSI analysis; there were very few differences between men and women gamblers in the same gambling domain (Table 5). No significant differences were observed between men and women for any of the PGSI-items in the chance-public domain. The hypothesis that women experience more gambling-related health problems was not supported in any domain. In addition, no support was found for the hypothesis that women in the domain of public domains experienced more criticism for their gambling or that women in these domains felt more guilt than men for their gambling. Thereby, hypothesis 4a was not supported.

**Table 5:** Proportions of men and women in each domain responding affirmatively to the nine PGSI-items and the significance according to Mann–Whitney rank test

	Chance-public (Proportion % and mean rank within gambling domain)		Strategy-domestic (Proportion % and mean rank within gambling domain)		Chance-domestic (Proportion % and mean rank within gambling domain)		Strategy-public (Proportion % and mean rank within gambling domain)	
PGSI Item	Men	Women	Men	Women	Men	Women	Men	Women
	*z* = −1.23 *p* = .222		*z* = −1.22 *p* = .221		*z* = −1.05, *p* = .293		***z*** **=** P**−2.47,** ***p*** **= .014**	
PGSI 1 Betted more than you can afford to lose?	87 (12.6, 482)	44 (15.7, 497)	93 (13.0, 401)	16 (17.8, 420)	113 (7.6, 1250)	89 (8.7, 1265)	**92 (10.9, 514)**	**34 (17.4, 547)**
	*z* = −1.32 *p* = .192		*z* = −1.32, *p* = .019		***z*** **= −2.92,** ***p*** **= .004**		*z* = −1.21, *p* = .228	
PGSI 2 Gambled with larger amounts?	89 (12.9, 490)	28 (10.0, 476)	100 (14.0, 405)	8 (8.9, 385)	**117 (7.8, 1271)**	**50 (4.9, 1233)**	110 (13.0, 524)	19 (9.7; 507)
	*z* = −.91, *p* = .360		***z*** **= −1.89,** ***p*** **= .050**		***z*** **= −2.08,** ***p*** **= .38**		***z*** **= −2.95,** ***p*** **= .003**	
PGSI 3 Tried to win back money?	126 (18.2, 490)	44 (15.7, 478)	**173 (24.2, 407)**	**13 (10.1, 370)**	**196 (13.1, 1270)**	**105 (10.3, 1235)**	**168 (19.9, 529)**	**20 (10.3, 482)**
	*z* = −.33, *p* = .074		*z* = −1.19, *p* = .234		*z* = −1.08, *p* = .280		*z* = −.21, *p* = .836	
PGSI 4 Borrowed money or sold anything to gamble?	46 (6.6, 488)	17 (6.1, 485)	36 (5.0, 404)	2 (2.2, 392)	51 (3.4, 1260)	27 (2.7, 1250)	51 (6.0, 521)	11 (5.6, 519)
	*z* = −.64, *p* = .520		*z* = −2.71, *p* = .790		*z* = −.99, *p* = .323		*z* = −.40, *p* = .687	
PGSI 5 Felt that you might have gambling problem?	20 (2.9, 487)	6 (2.1, 484)	13 (11.8, 403)	2 (2.2, 392)	16 (1.1, 1258)	7 (.7, 1253)	10 (1.2, 520)	3 (.5, 522)
	*z* = −1.04, *p* = .300		*z* = −.99, *p* = .321		*z* = −1.45, *p* = .147		*z* = −1.05, *p* = .290	
PGSI 6 Caused any health problems?	48 (6.9, 484)	25 (8.9, 493)	44 (6.2, 402)	8 (8.9, 405)	48 (3.2, 1250)	44 (4.3, 1264)	41 (4.9, 519)	13 (6.7, 528)
	*z* = −1.12, *p* = .240		*z* = .83, *p* = .404		*z* = −.50, *p* = .620		*z* = −.82, *p* = .409	
PGSI 7 Criticism against your gambling?	56 (8.1, 484)	29 (10.4, 495)	77 (10.8, 404)	7 (7.8, 392)	68 (4.6, 1258)	42 (4.1, 1253)	67 (7.9, 519)	19 (9.2, 528)
	*z* = .83, *p* = .404		*z* = −1.85, *p* = .064		*z* = −1.70, *p* = 1.09		***z*** **= −2.26,** ***p*** **= .024**	
PGSI 8 Caused any financial problems?	33 (4.8, 485)	17 (6.1, 491)	26 (3.6, 401)	7 (7.8, 418)	32 (2.1, 1250)	33 (3.2, 1264)	**30 (3.6, 517)**	**14 (7.2, 536)**
	*z* = −1.37, *p* = .171		*z* = −.51, *p* = .061		*z* = −1.22, *p* = .220		*z* = −.83, *p* = .407	
PGSI 9 Felt guilty about your gambling?	62 (9.0, 483)	33 (11.8, 497)	68 (9.5, 402)	10 (11.1, 409)	68 (4.6, 1251)	57 (5.6, 1264)	67 (7.9, 519)	19 (9.7, 528)

Bold figures indicate statistical significance

In the strategy-domestic domain, men were more likely than women to have gone back another day to try to win back money (PGSI 3). In addition, men were more likely to have gambled with larger amounts of money to feel the same excitement (PGSI 2) in all domains other than strategy-domestic. Despite this, hypothesis 4b; that men experienced more financial problems than women in any domain was not supported. In contrast, women in the strategy-public domain were more likely to have had financial problems caused by gambling than men. In addition, significantly more women than men in this domain reported they had gambled for more money than they could afford to lose.

## Discussion

The present study aimed to examine how different features of problem gambling presented in men and women who gambled regularly in Sweden, taking a domain approach based on gambling features and settings. In a gender equal society, men and women’s health behavior should come to resemble each other (Backhans et al. 2007). Research indicates that Sweden is country with a high degree of gender equality (Bambra et al. 2009). However, as noted, gender equality does not follow a modernistic idea of deterministic progress (Holdsworth et al. 2012). Men and women still gamble in different domains and to some extent, women seem to refrain from gambling in masculine domains and prefer the chance-domestic domain. As we predicted in hypothesis 1, more men than women participated in gambling domains associated with features of strategy and were overrepresented in gambling in public places. Whether this is due to gender socialization experiences, gender roles, discrimination or more intrinsic motivations within men and women, and how all these factors interact, is not known.

We expected that men who gambled regularly would have more problems with their gambling than women because they were more involved in domains associated with higher risk forms of gambling and their average age was lower. However, men and women who gambled regularly were just as likely to be problem gamblers. This finding is interesting in relation to a previous study, which showed that men bet more and gamble more frequently than women among regular gamblers (Wardle et al. 2011). However if controlling for age and gambling in multiple domains, women were actually more at risk than men for problem gambling. These findings suggest that women who gamble regularly may have a higher susceptibility to gambling problems, although they gamble less. However, our results are consistent with Clarke et al.’s (2006) findings that social, cultural and economic factors may explain why people start to gamble, while stress and loneliness predict the continuing of problem gambling. This could explain why women are less likely to be regular gamblers, especially in masculine gambling domains and why men dominate these domains. Research has shown that women in general report more stress and depression than men, which may be one factor explaining regular female gamblers vulnerablility to problem gambling (Statens folkhälsoinstitut 2012; Carlsson et al. 2013). However, further research is required to determine why women who do gamble regularly are at higher risk of problem gambling than men.

As hypothesized, gambling in the chance-domestic domain was associated with lesser degree of problem gambling but only for men who gambled regularly, not women. All other domains were associated with a higher likelihood of problem gambling for men in the bi-variate analysis, although this disappeared for the strategy-public and chance-public domains when controlling for age and gambling in other domains. The chance-public domain was the only domain associated with problem gambling for women in bi-variate as well as in multi-variate analyses, and the association became stronger when controlling for age and gambling in other domains while the association with problem gambling for men disappeared. Gambling appears to be a leisure activity that interacts with gender, with different behaviors and meaning for men and women. Gambling in the chance-public domain involved the use of gambling machines. This is consistent with findings from most published studies on problem gambling and gambling forms, which have shown that gambling machines are the form of gambling most associated with problem gambling (Delfabbro 2009; Moore et al. 2011; Williams et al. 2012). The strategy domains were only related to gambling problems for men who gambled regularly, not women. These domains included few women which may have affected the results reaching significance. However, this may be an indication that women who prefer strategy or skill based gambling (masculine-coded domains) and avoid chance or luck based gambling (feminine domains), may be seeking different goals from their gambling than women in other domains. This type of gambling could be a serious leisure activity that widens the gender roles and improves their self-image, as a way of negotiating femininity.

Furthermore, we found few differences between men and women in all domains regarding their scores on the nine PGSI-items. In the chance-public domain, no differences between men and women who gambled regularly were observed when examining each PGSI-item separately.

The effect of gambling in relation to the different PGSI-items within the same gambling domains was similar for men and women. Our prediction (Hypothesis 4a) that women in all domains would experience more health related problems (PGSI 6) was not supported by our results. In addition, hypothesis 4b was not supported since men did not experience more financial problems than women in any domain. It is important to note that not all gambling forms allow for increased bets, for example lotteries or bingo have maximum bet sizes and winnings. In contrast, women in the strategy-public domain were more likely than men to report that their gambling had caused them financial problems. One factor involved in this finding could be that women generally have lower incomes than men, making them more vulnerable to financial losses.

Problem gambling is still related to stigma, and this may especially be the case for women who experience additional concerns from failure to meet traditional caring responsibilities (McMillen et al. 2004). Our hypothesis that women experienced more criticism if they gambled in the public domains did first appear to be partly supported by the findings. However, these findings were not statistically significant. These results would have been consistent with previous studies on gambling in public and domestic places from a gender perspective (Hing and Breen 2001; Casey 2006).

We also noted that regular male gamblers were more likely than regular female gamblers to chase losses in every domain except chance-public. This PGSI-item (PGSI 3) was also the most endorsed one for men: a quarter of the men in the strategy-domestic domain had chased their losses. The most endorsed PGSI-item for regular female gamblers was betting more than they could afford to lose (PGSI 1).

The results are in some regards correspondent with other research. The most endorsed PGSI-item in the British Columbia Study (2009) was “gone back another day to try to win back the money you had lost”, 8 % of past-year gamblers. The least endorsed item at 1 % was that gamblers had borrowed money or sold anything to get money to gamble (Ipsos-Reid and Gemini Research 2008). Borrowing money was also the least endorsed item in the UK prevalence study, while the most endorsed item for both men and women was chasing losses: 6.9 % men; 2.5 % women, followed by betting more than you could afford to lose: 5.7 % men; 1.5 % women (Wardle et al. 2011).

Our study has some important limitations. First, few gamblers participate in only one domain. However, this factor was controlled for in the multivariate models. Second, a quantitative study, such as the current study, cannot clarify reasons and motives in a qualitative manner. The current study did not address what gambling means for the individuals engaged in the different domains or the context of their gambling, which are important to understand. These issues should be the scope of future studies.

The statistical power was low in some cases especially when we analyzed problem gambling in women in the strategy domains, where few women gamble. There were only 90 women in our sample who scored as problem gamblers (PGSI 3+). The low statistical power was one reason we merged moderate risk gamblers and problem gamblers in some analyses. There may have been methodological problems with merging moderate risk gamblers with problem gamblers, because problem gamblers experience problems, while moderate risk gamblers may only be at risk of developing future problems. However, we were interested in both moderate risk gamblers and problem gamblers for two reasons: 1) the category of problem gamblers was too small for gender analyses and 2) including PGSI 8+ would have led to a more pathological or medical approach.

A further methodological concern is the construction of the gambling domains, which was theory driven. The division into domains makes it harder to understand specific gambling forms (e.g. gambling machines and bingo in the chance-public domain). However, the domains made it possible to test the hypothesis on gender and the characteristics of strategy-chance and public-domestic. Even though the domains are theory driven, we performed an explanatory factor analysis to validate the theoretical framework with the empirical data. Overall, the factor analysis supported the categorization even if some of the casino games that we classified as chance-based gambling could also be categorized as strategy-based gambling. The factor analysis also revealed that three domains may work slightly better than four. In that case the two strategy domains would be merged into one.

This study builds on previous research by testing both dimensions for the first time. In addition, using the domain approach enabled statistical analyses. If we had investigated specific gambling forms, the data would have been too few to make gender comparisons. One dimension not addressed in the domain approach is the social aspect, such as if you gamble alone or with friends or family, which is an important dimension raised by Holtgraves (2009).

This study suggests that to avoid higher rates of problem gambling among women, more research into eventual effects of restrictions on access to gambling in the chance-public domain, including gambling machines and bingo, are required. Several restrictions regarding design, accessibility, and the supervision of age limits may be beneficial in the prevention of problem gambling. Researchers and policy makers should consider cultural aspects including how different gambling forms are perceived, designed and used in specific contexts. For example gambling machines differ significantly in their design and location depending on which country they are located in. Additionally, age and gambling domains (except chance-public), do not appear to play the same role for women as they do for men. This study provides increased understanding of how gambling behavior and preferences can result in problem gambling outcomes for women and men. While the differences between the genders are not large, they are present. Therefore, separate analyses for men and women are useful in unmasking gender inequality and differences, but the separation of men and women should be performed with careful consideration, not routinely, which could lead to cementing gender stereotypes. Our study shows that neither female nor male gamblers are homogenous groups, and it has implications for research and prevention of the negative effects of gambling.

## Appendicies. Key Measures in Swedish and English

### Appendix 1

**Speldomäner/regelbundet spelande [Gambling Domains/regular gambling]**: Det fanns en grindfråga för nio spelformer (hästspel, lotterier, nummerspel, bingo, sportspel, poker, kasinospel, spelautomater och att ringa in till livesända TV-tävlingar). Efter grundfrågan ställdes anpassade frågor om alla varianter av spelet. Grindfråga (exempel bingo): “Har du spelat bingo under de senaste 12 månaderna? Räkna inte med bingospel på spelmaskiner”. Svarsalternativ: “Ja” och “Nej”. Om respondenten svarade “Ja” på grindfrågan (här om bingo) fick personen följdfrågorna:

“Under de senaste 12 månaderna, hur ofta har du spelat på följande former av bingo?”:

- I en bingohall?
- Online på svenskaspel.se?
- Online på bingolotto.se?
- Online på något annat bolag?

Annat bingospel, som bilbingo?

Svarsalternativen var “Dagligen/nästan dagligen”, “Några gånger i veckan”, “En gång i veckan”, “Flera gånger i månaden”, “En gång i månaden”, 6–11 gånger per år” eller “Mer sällan/aldrig”.

- En variabel konstruerades för regelbundet spelande från svaren på alla varianter av spelformerna:
- *Domänen slumpspel i hemmiljö*: Harry Boy online, nätbingo, lotterier, Lotto (hos ombud eller online), nätkasino, spelmaskiner på nätet, ringt in till live-sända TV-tävlingar för pengar, skraplotter (på svenskaspel.se eller online hos andra bolag eller hos ett ombud).
- *Domänen strategispel i hemmiljö*: Hästspel på nätet, sportspel hos ombud eller på nätet och nätpoker.
- *Domänen slumpspel i offentlig miljö*: Bingo i bingohall, bilbingo, spelmaskiner (på kasino, bingohallar, internet, båtar eller pubar), Harry Boy hos spelombud, tombolalotter, kasinospel (på kasinon, pubar, båtar eller klubbar).
- *Domänen strategispel i offentlig miljö*: Hästspel på travbanor eller hos ombud, privat vadhållning och pokerspel på pubar, kasinon, privat eller på klubb.

There was one grid question for each of the nine gambling forms (horses, lotteries, number games, bingo, sports betting, poker, casino gambling, electronic gaming machines and betting money on live TV-shows). After the grid questions: the questions for each sub-mode were adjusted to the specific gambling form. Grid question (example for bingo): “Have you played bingo for money during the last 12 months? Don’t count bingo machines.” Answer format: “Yes” or “No”.

If the respondent answered “Yes” to the grid question (here on bingo):

“In the past 12 months, how often have you placed bets on the following types of bingo games:

- At a bingo hall?
- On-line bingo at svenskaspel.se?
- On-line bingo at bingolotto.se?
- On-line bingo with another gaming company?
- Other bingo games, for example, car bingo?”

The answer format in all types of gambling were “Daily/Almost daily”, “Several times/week”, “Once/week”, “Several times/month”, “Once/month”, “6–11 times/year”, “Less often/Never”

A variable for regular gambling was created from the results in all gambling modes according to following categories:

- *The chance-domestic domain included following gambling forms*: Harry Boy via internet, online bingo, lottery, Lotto (via gambling agent or online), online casino, online gambling machines, betting money on live TV-shows, scratch tickets (online at Svenska Spel or online at other companies or at a gaming agent).
- *The strategy-domestic domain included following gambling forms*: Horses via internet, sports betting (via gambling agents or online) and online poker.
- *The chance-public domain included following gambling forms*: Bingo hall, car bingo, gambling machines (at casinos; bingo halls, boats or pubs), Harry Boy at gambling agent, lottery (tombola); casino gambling (at casinos, pubs, boats or clubs).
- *The strategy-public domain included following gambling forms*: Horses at racing tracks, horses at gambling agent, private sports betting, poker (at casinos, private clubs, or clubs)

### Appendix 2

**Problem Gambling Severity Index (PGSI)**: PGSI består av nio frågor som mäter spelproblem och risk för spelproblem de senaste 12 månaderna. Svarsalternativen är “Aldrig”, “Ibland”, “ofta” och “nästan alltid”.

1. Har du spelat för mer än du verkligen haft råd att förlora?
2. Har du behövt spela med större summor för att få samma känsla av spänning?
3. Har det hänt att du återvänt någon annan dag för att vinna tillbaka det du förlorat?
4. Har du lånat pengar eller sålt något för att ha pengar att spela för?
5. Har du känt att du kanske har problem med ditt spelande?
6. Har spelandet orsakat dig några problem med din hälsa, inräknat stress eller ångest?
7. Har någon kritiserat ditt spelande eller sagt att du har problem med spelandet, oavsett om du tyckt det varit sant eller inte?
8. Har ditt spelande orsakat några ekonomiska problem för dig eller ditt hushåll?
9. Har du känt skuld över hur du spelar, eller vad som händer när du spelar?

Svaren på de nio frågorna ges följande poäng: Aldrig = 0; Ibland = 1; Ofta = 2; Nästan alltid = 3 och summeras. Maxpoäng är 27. 0 = inga spelproblem, 1–2 = viss risk för spelproblem, 3–7 = moderat risk för spelproblem och 8 eller mer = spelproblem

I Swelogs används en alternativ indelning i tre olika kategorier:

- 0 = Inga spelproblem
- 1–2 = Viss risk
- 3 eller mer = Problemspelande

Problem gambling Severity Index include 9 questions regarding the last 12 months with answer format “Never”, “Sometimes”, “Most of the time”, “Almost always”.

1. Have you bet more than you could really afford to lose?
2. Still thinking about the last 12 month, have you needed to gamble with larger amounts of money to get the same feeling of excitement?
3. When you gambled, did you go back another day to try to win back the money you lost?
4. Have you borrowed money or sold anything to get money to gamble?
5. Have you felt that you might have a problem with gambling?
6. Has gambling caused you any health problems, including stress or anxiety?
7. Have people criticized your betting or told you that you had a gambling problem, regardless of whether or not you thought it was true?
8. Has your gambling caused any financial problems for you or your household?
9. Have you felt guilty about the way you gamble or what happens when you gamble?

Score the following for each response: Never = 0, Sometimes = 1, Most of the time = 2, Almost always = 3

Scores for the 9 items are summed with a maximum score of 27. The results are interpreted as follows: 0 = Non-problem gambling. 1–2 = Low risk of problems with few or no identified negative consequences. 3–7 = Moderate risk for problems leading to some negative consequences. 8 or more = Problem gambling with negative consequences and a possible loss of control.

In Swelogs “Moderate risk” and “problem gambling” was merged into one group called problem gambling.
